# Obsessive Compulsive Personality Disorder and Parkinson’s Disease

**DOI:** 10.1371/journal.pone.0054822

**Published:** 2013-01-24

**Authors:** Alessandra Nicoletti, Antonina Luca, Loredana Raciti, Donatella Contrafatto, Elisa Bruno, Valeria Dibilio, Giorgia Sciacca, Giovanni Mostile, Antonio Petralia, Mario Zappia

**Affiliations:** 1 Dipartimento “G.F. Ingrassia” Sezione di Neuroscienze, Università di Catania, Catania, Italy; 2 Dipartimento di Biomedicina Clinica e Molecolare, Sezione di Psichiatria, Università di Catania, Catania, Italy; Institute of Psychiatry at the Federal University of Rio de Janeiro, Brazil

## Abstract

**Objectives:**

To evaluate the frequency of personality disorders in Parkinson’s disease (PD) patients and in a group of healthy controls.

**Methods:**

Patients affected by PD diagnosed according to the United Kingdom Parkinson’s disease Society Brain Bank diagnostic criteria and a group of healthy controls were enrolled in the study. PD patients with cognitive impairment were excluded from the study. Structured Clinical Interview for Personality Disorders-II (SCID-II) has been performed to evaluate the presence of personality disorders. Presence of personality disorders, diagnosed according to the DSM-IV, was confirmed by a psychiatric interview. Clinical and pharmacological data were also recorded using a standardized questionnaire.

**Results:**

100 PD patients (57 men; mean age 59.0±10.2 years) and 100 healthy subjects (52 men; mean age 58.1±11.4 years) were enrolled in the study. The most common personality disorder was the obsessive-compulsive personality disorder diagnosed in 40 PD patients and in 10 controls subjects (*p-value*<0.0001) followed by the depressive personality disorder recorded in 14 PD patients and 4 control subjects (*p-value* 0.02). Obsessive-compulsive personality disorder was also found in 8 out of 16 *de novo* PD patients with a short disease duration.

**Conclusion:**

PD patients presented a high frequency of obsessive-compulsive personality disorder that does not seem to be related with both disease duration and dopaminergic therapy.

## Introduction

Since at least 1913, several reports have evaluated the association between personality traits and Parkinson’s disease (PD), generally suggesting a personality profile characterized by industriousness, inflexibility, punctuality, cautiousness and lack of novelty seeking [1]–[2].

More recently, several studies have investigated personality among PD patients considering the psychobiological model of temperament and character [3]. According to Cloninger’s model, temperament traits (novelty seeking, harm avoidance and reward dependence) are related to brain systems modulated by dopamine, serotonin, and noradrenaline, respectively. A reduced novelty seeking and increased harm avoidance among PD patients have been consistently reported [2] and some studies have shown that the degree of reduced novelty seeking in PD patients was correlated with specific patterns of impaired dopamine functioning. [4] On the bases of these evidences a “premorbid” parkinsonian personality has been suggested and it has been hypothesized that it could be a possible early manifestation of the neurological changes in the brain. However, despite several case-report, case series, twin studies, and case-control studies support this hypothesis, to date the idea of such a distinctive premorbid personality profile remains controversial [2].

To the best of our knowledge, even if parkinsonian personality has been largely investigated, no studies have systematically evaluated the presence of Personality Disorders (PeDs) defined according to the Diagnostic and Statistic Manual of Mental Disorders (DSM-IV) in PD patients. PeDs (noted on Axes II), formerly referred to as character disorders, are a class of personality types and behaviours defined as “an enduring pattern of inner experience and behaviour that deviates markedly from the expectations of the culture of the individual who exhibits it” [5]. According to the DSM-IV we distinguished: paranoid, schizoid, schizotypal, antisocial, borderline, histrionic, narcissistic, avoidant, dependent, obsessive compulsive, depressive and passive aggressive PeDs [5].

We evaluated the presence of PeDs, according to the DSM-IV criteria, in a large sample of PD patients and in a group of healthy subjects.

## Materials and Methods

Patients affected by PD diagnosed according to the United Kingdom Parkinson’s disease Society Brain Bank diagnostic criteria [6] were consecutively enrolled in the study during the period April 2010– October 2010 from the Movement Disorders Center of the University of Catania. Healthy controls with no neurological or psychiatric disorders were recruited from 10 randomly selected general practitioners rosters in the Province of Catania. Control subjects were group matched by sex and age. For PD subjects, clinical evaluation of motor status was made using the Hoehn and Yahr [7] stage and the Unified Parkinson’s Disease Rating Scale-Motor Examination section (UPDRS-ME) [8]. The UPDRS-ME scores were recorded while the patients were in “on” motor status. In order to evaluate the possible presence of cognitive impairment, the Mini Mental State Examination (MMSE) was administered and PD patients with a MMSE score lower than 24 were excluded [9]. The study was approved by the local ethical committee and patients and controls were enrolled only after signed the informed consent.

To diagnose the presence of PeDs we adopted the widely used Structured Clinical Interview for DSM-IV Personality Disorders (SCID-II) and the associated SCID-II Personality Questionnaire (SCID-II-PQ) [10]. The SCID-II have been constructed so as to be conceptually and methodologically related and tightly linked to DSM criteria. In the typical use of the SCID-II assessment approach, the examined first completes a self-report questionnaire (the SCID-II-PQ); positive items are then further explored in a semi-structured diagnostic interview (the SCID-II) performed by a psychiatrist. The combined use of a self-rating screening tool together with the interview has been reported to have good validity for axis II diagnosis [11].

The SCID-II, is not a disease-related instrument and it has been already used to investigate PeDs in several neurological disorders including PD and essential tremor [12]–[14].

PeDs were diagnosed according to the DSM-IV criteria [5]. According to DSM-IV personality disorders the ten PeDs are grouped into three main clusters: cluster A or “odd-eccentric” disorders (paranoid, schizoid, schizotypal); cluster B or “dramatic-emotional” disorders (antisocial, borderline, histrionic, narcissistic); and cluster C or “anxious-fearful” disorders (avoidant, dependent, obsessive-compulsive) [5].

Data were analyzed using STATA 10.0 software packages [15]. Quantitative variables were described using mean and standard deviation. The difference between means and the difference between proportions was evaluated by the t-test and the Chi-square test respectively. In case of not a normal distribution appropriate non-parametric tests were performed. Unconditional logistic regression analysis was performed and odds ratio (OR), 95% Confidence Interval (CI), and *p* values (two-tailed test, p  = 0.05) were calculated. In order to evaluate the possible relationship between clinical parameters and presence of PeDs univariate and multivariate analyses were performed only for PD patients. Presence of PeD was considered as outcome variable. Unconditional logistic regression analysis was performed and for each clinical variable (treatment, age at onset, UPDRS-ME etc.) we calculated OR, 95% CI, and p-value (two-tailed test, p  = 0.05). Whenever variables were dichotomized the cut-offs were derived from the median value of the pooled distribution of cases.

## Results

One-hundred and three PD patients were eligible for the study. Three of them refused to participate. At the end of the study, we enrolled 100 PD patients (57 men and 43 women; aged 59.0±10.2 years) and 100 healthy subjects (52 men and 48 women; aged 58.1±11.4 years). No significant differences in age, sex and years of schooling (9.8±3.6 years among cases *versus* 9.3±4.6 years among controls) have been recorded between cases and controls. The mean age at onset among PD patients was 54.8±10.8 years with a mean disease duration of 5.0±4.0 years. The UPDRS-ME mean score was 26.7±12.2 and the mean Hoehn and Yahr stage was 1.9±0.6 (ranging from 1 to 3).

Out of the 100 PD patients 12 (6 men and 6 women) had had the onset of the disease before the age of 40 years (early onset). Concerning antiparkinsonian treatment at the time of evaluation, 53 patients were taking L-dopa (LD) alone; 19 LD in combination with dopamine agonists (DAs); seven DAs alone while five were taking other type of antiparkinsonian treatment. Sixteen PD patients were *de novo*, never treated with antiparkinsonian agents.

Out of the 100 interviewed PD patients, 80 fulfilled the DSM-IV diagnostic criteria for the presence of at least one PeDs (67 patients presented only one PeD and 13 more than one PeD), while only 24 control subjects were affected by at least one PeD (20 subjects presented only one PeD and 4 more than one PeD). Frequency of PeDs in both PD patients and controls is shown in table 1.

**Table 1:** Frequency of PeDs among PD patients and controls.

PeDs	PD(n = 100)	Controls(n = 100)	*p-value*
Avoidant	6	1	**0.05**
Dependent	3	/	0.08
Obsessive Compulsive	32	7	**<0.0001**
Passive aggressive	1	1	1.0
Depressive	11	4	**0.003**
Paranoid	4	5	0.7
Schizotypal	1	1	1.0
Schizoid	1	/	0.3
Histrionic	3	/	0.08
Narcissistic	2	/	0.1
Borderline	3	/	0.08
Antisocial	/	1	0.1
More than one	13*	4**	0.02

PeDs =  Personality Disorders;

* = 8 were affected by obsessive-compulsive PeD and 3 by Depressive PeD;

** 3 were affected by obsessive-compulsive PeD.

According to the DSM-IV classification obsessive-compulsive PeD (OCPeD) was the commonest disorder being recorded in 40 PD patients (32 among patients presenting only one PeD and 8 out of the 13 patients presenting more than one PeD) and in 10 control subjects (7 among subjects presenting only one PeD and 3 out of the 4 subjects presenting more than one PeD) giving an OR of 6.0 (95% CI 2.79–12.9; p-value <0.0001). OCPeD was significantly more frequent among PD patient aged 60 years and above (OR 4.24, *p-value* 0.001), while it was not significantly associated with UPDRS-ME score, treatment and early onset as shown in table 2. OCPeD was more frequent among men (45.6%) than women (32.6%) even if such difference was not significant (p-value 0.2). Multivariable analysis did not change the strength of the association found at the univariate analysis.

**Table 2:** OCPeD, Depressive PeD and clinical characteristics.

	OCDeP				Depressive PeD			
	N (%)	OR	95%CI	p-value	N (%)	OR	95%CI	p-value
**Age**								
≤60	11 (22.9)	1			10 (20.8)	1		
>60	29 (55. 8)	4.24	1.78–10.1	**0.001**	4 (7.7)	0.31	0.09–1.08	0.07
**Early onset**								
≤40	2 (16.7)	1			2 (16.7)	1		
>40	38 (43.2)	3.8	0.79–18.4	0.1	12 (13.6)	0.79	0.15–4.05	0.8
**sex**								
Women	14 (32.6)	1			10 (23.3)	1		
Men	26 (45.6)	1.73	0.76–3.96	0.2	4 (7.0)	0.24	0.07–0.85	**0.02**
**UPDRS-ME**								
≤23	21 (38.9)	1			5 (9.3)	1		
>23	19 (41.3)	1.10	0.49–2.47	0.8	9 (19.6)	2.38	0.73–7.71	0.1
**Treatment**								
None	8 (50.0)	1			2 (12.5)	1		
LD	20 (37.7)	0.60	0.20–1.87	0.3	7 (13.2)	1.06	0.20–5.72	0.07
DA	1 (14.3)	0.16	0.02–1.72	0.1	2 (28.6)	2.8	0.31–25.52	0.9
LD +DA	9 (47.4)	0.9	0.24–3.41	0.9	3 (15.8)	1.31	0.19–9.02	0.3
others	2 (40.0)	0.67	0.09–5.13	0.7	0 (0)	/	/	/

**Figure 1:**
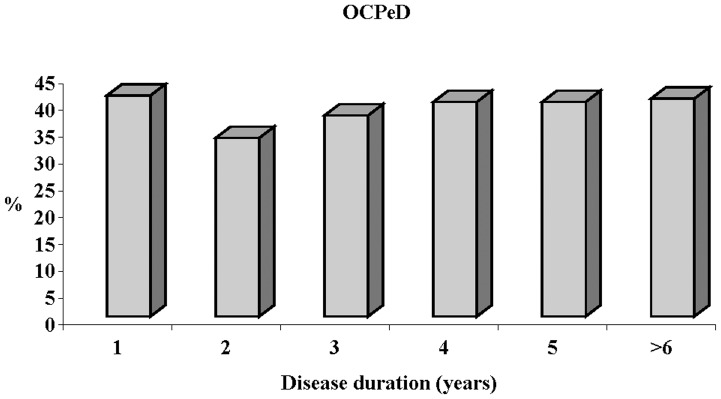
Frequency OCPeD by disease duration. OCPeD = Obsessive-compulsive Personality Disorder.

LD = Levodopa; DA =  Dopamine Agonist;

OCPeD was present in 8 (50%) out of the 16 *de novo* PD patients enrolled in the study (disease duration 1.9±2.3 years). Frequency of OCPeD seems to be independent of disease duration remaining stable over the time as shown in figure 1.

A lower frequency of OCPeD (16.7%) was recorded among the 12 PD patients with an early onset (<40 years). Nevertheless it should be noted that all these 12 patients presented at least one PeDs.

Depressive PeD was the second most frequent disorder recorded in 14 PD patients (11 among patients presenting only one PeD and 3 out of the 13 patients presenting more than one PeD) and in four control subjects (OR  = 3.91; 95% CI 1.23–12.3; p-value 0.02). Depressive PeD was significantly more frequent among women (p-value 0.02), while it was not significantly associated with UPDRS-ME score, age, treatment and early onset (table 2).

As shown in table 1 the majority of PeDs recorded among PD patients were those included in cluster C (“anxious-fearful” disorders), while only few patients presented a PeDs of cluster A (“odd-eccentric”) or cluster B (“dramatic-emotional”).

## Discussion

Even if several studies have been carried out to evaluate temperament and personality traits among PD patients, PeDs, defined according to the DSM-IV, have been scarcely investigated. PeDs represent a quite common condition in the general population, and in particular prevalence of OCPeD in the general population has been estimated to be around of about 8.0% in a very large population survey recently carried out [16]. In agreement with this data we found a close frequency of OCPeD in our control group (10%) [16].

Although most patients with a PeD do not require specific treatment, some more severe cases may improve with pharmacological and/or psychotherapeutic treatment [17]. On these grounds, our survey firstly has investigated the prevalence of PeDs defined according to the DSM-IV in a PD population. Overall we found an higher frequency of PeDs in PD patients respect to a control group, mainly due to a very high frequency of OCPeD among PD patients (40%) and, in part, also to a higher frequency of depressive PeD (14% in PD patients and 4% in controls subjects).

According to the DSM-IV, obsessive compulsive personality disorder (OCPeD) is defined as a pervasive pattern of preoccupation with orderliness, perfectionism, and mental and interpersonal control at the expense of flexibility, openness, and efficiency [5]. It should be underlined that the characteristics of OCPeD, as defined by the DSM-IV criteria, appear to overlap with the “parkinsonian personality” consistently reported in literature over the time [1]–[2]. Our data suggest that such characteristics could be considered not just a trait, as it has been commonly described, but as a well defined disorder that could be diagnosed according to worldwide accepted diagnostic criteria.

Literature data have also suggested a possible increased frequency of Obsessive-Compulsive Disorders (OCD, noted on Axes I according to the DSM-IV) among PD patients, but to date, probably due to methodological differences across the studies, this association remains controversial [18]. On the other hand also the relationship between OCD and OCPeD has been a subject of interest and controversy. OCPeD, in fact, has been regarded as a precursor to OCD, but to date, even if there are some overlap between these two conditions, how closely they are remains uncertain [19]. PD is characterized by dysfunction in the fronto-basal ganglia circuitry and a similar circuitry has also been implicated in the pathophysiology of OCD. It is believed that the higher incidence of obsessive-compulsive symptoms in PD is due to the involvement of a shared circuitry [20].

In agreement with some literature evidences in our sample, frequency of OCPeD was significantly higher among PD patients aged 60 years or above. Prevalence studies addressing specific personality disorders, in fact, have shown that within different subpopulations personality pathology of clusters C, such as OCPeD, are quite common in older adults [21]. It should be underlined that, although personality disorders have historically been considered as stable over time, clinical presentation of these disorders may change across the life-time including in the elderly. Personality disorders, in fact, may attenuate, re-emerge or appear de novo according to the social context. [22].

Concerning the other PeDs, the second most common disorder in our PD population was the Depressive PeD. Depressive personality disorder is a controversial psychiatric diagnosis that denotes a personality disorder with depressive features. Depressive personality disorder was added to DSM-IV’s appendix B (Criteria Sets and Axes Provided for Further Study) amid controversy and the main issue was whether it could be differentiated conceptually and empirically from dysthymia and major depression [23]. Considering the well known high prevalence of depression among the PD patients, we are aware that in some case distinguishing between these two conditions can be difficult.

Finally also avoidant PeD (characterized by a pervasive feelings of social inhibition and social inadequacy, extreme sensitivity to negative evaluation and avoidance of social interaction) and dependent PeD (characterized by a pervasive psychological dependence on other people) were more common among PD patients than controls. According to the DSM-IV [5] both avoidant and dependent personality disorders, along with the obsessive-compulsive one, are grouped in the cluster C. Even if the few number of events in these categories do not allow us to exclude the possible role of chance, the possible association between PD and both avoidance and dependent PeD could be explained by an high level of harm avoidance, that according to Cloninger’s model [3], is the temperament trait characterizing PD patients [2]. A recent study, in fact, has highlighted the relationships between dimensional and categorical approaches of personality showing a positive correlations between harm avoidance and cluster C personality disorders [24].

We are aware that common limits of cross-sectional studies should be taken into account in interpreting our analyses. Selection of cases and controls generally represents the most important pitfall in case-control studies. As in several of case-control studies, also in our study PD patients were enrolled from a hospital setting. Cases selected from a hospital setting could include more severely affected patients not representing the total parkinsonian population and possibly resulting in a reduced generalization. However in our sample the Hoehn-Yahr stage (1.9±0.6) revels a mild or moderate stage of disease. Moreover we cannot be sure that the patient’s personality traits were evident before the onset of PD, in young adulthood and endured throughout life. To this reason, as recommended by the SCID-II guideline, subjects were specifically instructed to answer considering their entire life since young adulthood. Furthermore, during the interview, for each positive item of the SCID-II questionnaire, the examinator specifically asked if that behaviour was present since the adolescence [10]. However the possible bias in recall is a common limit related to the majority of the studies carried out to investigate the parkinsonian personality [2]. To the best of our knowledge, in fact, only one recent historical cohort study has been performed to investigate the possible premorbid personality in PD [25]–[26]. At any rate, even if we cannot be sure that this condition was already present during the adolescence, due to the high prevalence of OCPeD found in the *de novo* PD patients and in general in patients with a short disease duration as shown in figure 1, it could be probably considered an early manifestation of PD. Nowadays great attention is focused on the identification of possible markers of the early stage of the disease. Many non-motor symptoms occur early in PD and some of them, such as olfactory deficit, REM behaviour disorder, depression, constipation, may even predate also of many decades [27] the diagnosis of PD which is based on motor signs [28]. Nevertheless to confirm that PeDs could precede PD onset, prospective researches are required, even if defining the true premorbid time period could be impossible without valid biomarkers to reliably detect PD before the emergence of motor symptoms. Studies on a larger population of *de novo* PD patients at early stage of disease are needed to support our findings.
